# Relationship Between the Preferred Place of Death in Patients’ Health Records and the Actual Location of Death in Home Palliative Care: A Retrospective Cohort Study

**DOI:** 10.3390/healthcare14081043

**Published:** 2026-04-15

**Authors:** Almudena Castaño Reguillo, Raquel Sánchez Ruano, Jaime Barrio Cortés, Elena Polentinos-Castro, ESAPD Espronceda Collaborative Group

**Affiliations:** 1 Family and Community Medicine, CS Los Angeles, Madrid Health Service (SERMAS), 28021 Madrid, Spain; 2 Doctoral Program in Epidemiology and Public Health (Interuniversity), Rey Juan Carlos University, 28032 Madrid, Spain; rsruano@salud.madrid.org; 3 Research Unit, Primary Care Assistance Management, Madrid Health Service (SERMAS), 28035 Madrid, Spain; elena.polentinos@salud.madrid.org; 4 Research Network on Chronicity, Primary Care and Health Promotion-RICAPPS-(RICORS), Instituto de Salud Carlos III (ISCIII), 28003 Madrid, Spain; 5 Gregorio Marañón Health Research Institute, Madrid Health Service (SERMAS), 28007 Madrid, Spain; 6 Foundation for Biosanitary Research and Innovation in Primary Care of the Community of Madrid (FIIBAP), 28003 Madrid, Spain; jaime.barrio@salud.madrid.org; 7 HM Faculty of Health Sciences, University Camilo José Cela, Castillo de Alarcón, 49, 28692 Madrid, Spain; 8 HM Hospitals Research Institute, 28692 Madrid, Spain; 9 Department of Medical Specialties and Public Health, Rey Juan Carlos University, 28922 Alcorcón, Spain

**Keywords:** end-of-life care, terminal care, death, home patient-centred care, hospice, hospital, palliative care, place of death, preferences, advance care planning

## Abstract

**Background:** Dying in the preferred place is considered an indicator of the quality of end-of-life care. Advance care planning and home palliative care may increase the likelihood of dying at home, but it remains unclear whether the documentation of these preferences in clinical records is associated with the actual place of death. This study assessed whether the recording of patients’ preferred place of care or death was associated with the actual place of death among patients followed by home palliative care teams. **Methods:** We conducted a retrospective observational study with an exploratory approach that included adult patients who died in 2022 and were followed by a home palliative care team in Madrid, Spain. Data were extracted from electronic health records and analysed using bivariate tests and multivariable logistic regression. **Results:** A total of 464 patients were included (53% women; mean age 80.8 years). Overall, 82.5% of the patients died at home. Preferences were recorded for 64% of the patients; 97.6% preferred to die at home, and 89% died there. Documentation of preferences was not significantly associated with the place of death. **Conclusions:** Documentation alone was not independently associated with the place of death. These findings should be interpreted cautiously given the observational and exploratory design of the study and the limited explanatory power of the regression model. Notably, most patients expressed a preference to die at home, and most deaths also occurred at home. Recording preferences may still support advance care planning and patient-centred decision-making, but documentation should not be interpreted as sufficient in itself to influence place of death.

## 1. Background

Most people prefer to die at home when facing advanced or life-limiting illness [1,2,3]. However, in Spain and in other countries with similar socioeconomic characteristics, most deaths still occur in hospitals or institutional settings, while only approximately one-third occur at home [4,5,6,7,8]. Dying in the preferred place is considered an indicator of the quality of end-of-life care and a key component of a “good death” [9,10]. Recent umbrella reviews and updated evidence syntheses confirm that although home remains the most commonly preferred place of death, the achievement of this preference varies substantially across healthcare systems and depends on the availability of social support, caregiver involvement and community resources [11,12].

Previous studies have shown that the probability of dying at home is influenced by multiple clinical and social factors, including older age [5,7,8,13], the presence of a primary caregiver [14,15,16], functional decline [13,14], socioeconomic status [17,18], rural residence [7,15], marital status [5,7] and sex, although findings regarding sex remain inconsistent [6,7,13,19]. Recent studies suggest that many of these variables act as mediators between patient preferences and actual outcomes, indicating that expressing a preference alone does not necessarily guarantee that it will be honoured [20,21].

Home palliative care (HPC) services play a central role in enabling patients to remain at home at the end of life. Their involvement is associated with fewer emergency department visits and a lower probability of dying in a hospital [3,14,18,22,23]. Similarly, primary care follow-up and the availability of social and healthcare resources increase the likelihood of dying at home [13,15,24,25]. More recent cohort studies also suggest that early referral to specialist palliative care and longer follow-up duration may improve the concordance between the preferred and actual place of death, reinforcing the importance of continuity and coordinated care [26,27].

In this context, advance care planning (ACP) is particularly relevant. ACP is defined as a process that supports patients in understanding and sharing their values, goals and preferences regarding future medical care [28,29]. ACP has been shown to be associated with reduced hospital deaths and increased home deaths [23,30]. One of the most accessible components of ACP in routine practice is the documentation of a patient’s preferred place of death. However, ACP should not be understood as a single conversation but as an ongoing process that includes communication, documentation and accessibility of preferences across care settings. One of the most feasible and measurable components of ACP in routine clinical practice is the documentation of a patient’s preferred place of care or death in the clinical record. Recent evidence indicates that ACP interventions, including the clear and accessible documentation of preferences, are associated with higher concordance between the preferred and actual place of death [11,31].

Importantly, recorded preferences may not fully represent patients’ true or current wishes, as preferences can evolve over time or may be incompletely documented. Nevertheless, documentation in clinical records may act as a proxy for ACP activation, reflecting that goals-of-care conversations have occurred and enabling communication and coordination among professionals and caregivers [31,32]. In this sense, documentation should not be viewed solely as an administrative act but as an operational element of ACP that may influence end-of-life outcomes.

Although several studies have examined factors associated with place of death, few have specifically evaluated whether recording the preferred place of death in the clinical record is associated with the actual place of death among patients receiving home palliative care [32]. This represents an important scientific gap, as documenting preferences may reflect the existence of ACP discussions and may facilitate shared decision-making and coordinated care [33]. Furthermore, existing evidence has largely focused on stated preferences, whereas less is known about whether routine clinical documentation of these preferences is associated with measurable differences in end-of-life outcomes beyond patient characteristics alone.

By focusing on documentation as an operational component of ACP, this study explores whether the routine recording of preferences in clinical practice is associated with concordance between the preferred and actual place of death. The primary objective of this study was to analyse whether the explicit recording of the preferred place of care or death among patients followed by a home palliative care team is associated with a greater probability of dying in the preferred place. The secondary objectives were to explore other sociodemographic and clinical factors associated with the place of death.

## 2. Methods

### 2.1. Study Design and Setting

We conducted a retrospective observational cohort study that included patients followed by a home palliative care (HPC) team in Madrid, Spain. This study was exploratory in nature and aimed to identify associations in routinely collected clinical data rather than to establish causal relationships. The HPC team ESAPD Centro provides home-based palliative care to an urban population of approximately 671,000 inhabitants with diverse socioeconomic characteristics [34].

Home palliative care in Madrid is delivered by multidisciplinary teams integrated into the public primary care system, which provides universal coverage and free access at the point of care. These teams work in coordination with primary care services and specialized palliative care units.

In the Community of Madrid (Spain), home palliative care is integrated within the publicly funded National Health System and can be requested for any patient requiring this level of care, as healthcare coverage is universal and free at the point of access. Therefore, the variability in access to home palliative care services within the study area is expected to be limited.

The study was conducted and reported in accordance with the Strengthening the Reporting of Observational Studies in Epidemiology (STROBE) guidelines.

### 2.2. Study Population

The study population comprised all adult patients (≥18 years) followed by the HPC team who died in 2022. The inclusion criteria were active follow-up by the team and at least one face-to-face home visit.

Patients were excluded if the place of death was not recorded, if death occurred under special circumstances (e.g., euthanasia), or if discrepancies in death-related data could not be resolved after data verification.

### 2.3. Sample Size and Sampling

**Figure 1:**
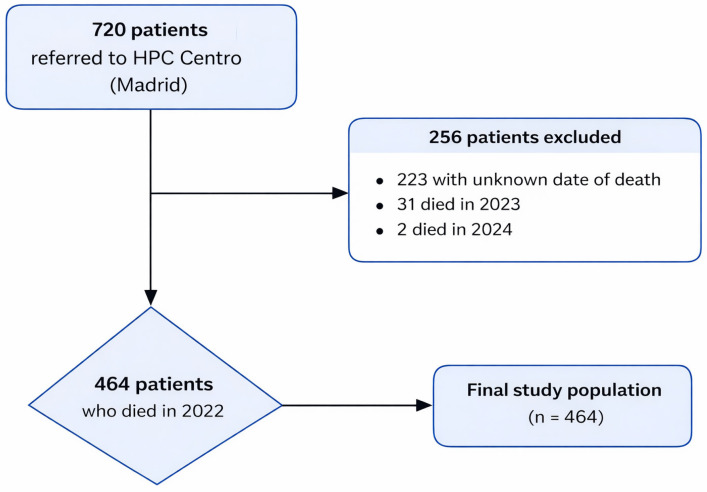
Flowchart of eligible participants.

No formal sample size calculation was performed, as all patients who met the eligibility criteria during the study period were included (n = 464). As this was a retrospective observational study based on routinely collected data, the available population determined the sample size; therefore, no a priori type I error, type II error or statistical power calculation was performed. The flowchart of participant eligibility is shown in Figure 1.

### 2.4. Data Source and Variables

Data were extracted from the electronic medical records system (“AP-Madrid”), which includes a specific palliative care protocol completed by healthcare professionals during patient assessment and follow-up. Sociodemographic variables (sex, age, presence of a primary caregiver, socioeconomic quartile of the area of residence), clinical variables (type of advanced disease (oncological or non-oncological) and number of weeks spent in the program (HPC follow-up duration), and information related to death (patients’ and their families’ preferred place of death and care (home/health centre), changes in place of death and care preferences, and actual place of death (home/health centre)) were extracted.

The main exposure variable was the recorded preferred place of care or death documented in the clinical record. For analytical purposes, we distinguished between the presence or absence of documentation of preferences and the content of the documented preference. The primary outcome was the actual place of death (home vs. non-home). Although the preferred place of care and preferred place of death are conceptually distinct, in this study, they were analysed together because of the structure of routine clinical records, which frequently combine both concepts within a single record.

### 2.5. Statistical Analysis

Continuous variables are expressed as medians and interquartile ranges (IQRs, 25–75%) or means and standard deviations (SDs). Categorical data are expressed as absolute and relative frequencies.

Descriptive analyses were performed to summarize sociodemographic, clinical and preference-related characteristics, with relative and absolute frequencies and measures of central tendency and dispersion reported according to the distribution of the data for the variable. Bivariate analyses were performed to assess associations between the place of death and sociodemographic, clinical and preference-related variables. Differences in variables between patients depending on the place of death were examined using chi-square tests for categorical variables and independent-samples t tests for continuous variables (or the Welch test when assumptions of normality were not fulfilled).

A logistic regression model was performed to adjust for other variables. Variables were selected for a multivariable model on the basis of clinical relevance and prior evidence, regardless of their statistical significance in bivariate analyses, to identify factors independently associated with death at home. The results are reported as adjusted odds ratios (aORs) with 95% confidence intervals (95% CI). Covariates were selected a priori on the basis of clinical relevance and previous literature; therefore, no univariable *p* value screening was used for variable selection, and all prespecified covariates were included in the multivariable model regardless of statistical significance. Hosmer–Lemeshow test was used to appraise the fit of the model, and also Nagelkerke’s R2 to evaluate performance of the model. Statistical significance was defined as *p* < 0.05.

Given the observational and exploratory nature of the study, a regression model, rather than a predictive model, was designed to identify associations; therefore, extended model performance analyses (e.g., discrimination or calibration statistics) were not the primary focus.

All analyses were performed using IBM SPSS Statistics version 26.0.

## 3. Results

### 3.1. Patient Characteristics

A total of 464 patients were included; 53% were women, and the mean age was 80.8 years (SD ± 13.0). The median duration of follow-up by the HPC team was 2.86 weeks (IQR 1–8.14), and 24.1% of patients had a recorded primary caregiver.

With respect to diagnosis, 61% of patients had oncological disease, most frequently digestive system cancers. Among the non-oncological conditions, heart failure was the most common diagnosis (Appendix A.1).

Overall, 82.5% of patients died at home, 14.1% died in a hospital, and 3.4% died in a mid-stay palliative care unit.

A preferred place of death was recorded for 24.6% of patients, whereas a preferred place of care or death was recorded for 64.0%. Among patients with a recorded preference, 97.6% expressed a preference for home, and 2.4% expressed a preference for a non-home setting. Among those who preferred home, 89.0% died at home.

Baseline characteristics and preferred and actual places of death are shown in Table 1.

### 3.2. Factors Associated with Place of Death

In the bivariate analysis, patients who died at home were older than those who died in institutional settings (mean difference: 3.8 years; *p* < 0.05). The percentage of patients who died at home was higher for women than men (86.2% vs. 78.4%, *p* < 0.05) (Table 2).

Among the patients, 87.2% of non-oncology patients and 79.6% of oncological patients died at home (*p* < 0.05). No significant differences were observed according to the presence of a primary caregiver.

Patients with a recorded preference for home more frequently died at home than did those without a recorded preference (89.5% vs. 80.3%, *p* = 0.024).

### 3.3. Factors Associated with Recording Preferences and Preferences Expressed

The study examined which factors might be associated with patients having their preferences recorded, finding that older people, not having a primary caregiver, and non-cancer patients were less likely to have their preferences recorded than cancer patients. Gender and area of residence were not associated with having these preferences recorded (Appendix A.2).

With respect to preferences for home as the place of death or end-of-life care, the bivariate analysis, only sex was associated with preference, showing that 99.4% of women preferred to die or receive care at home, compared to 95.8% of men (*p* = 0.042). Age was not statistically associated with preference for home as place of care/death (*p* = 0.661). When analyzed by type of condition, 99.0% of non-cancer patients and 96.9% of cancer patients selected home as their preferred place (*p* = 0.251). Regarding area of residence, home was chosen as the preferred location for care and death in both the most deprived (96.3%) and least deprived (97.6%) areas (*p* = 0.522). Similarly, regardless of whether a primary caregiver was registered, 98.0% and 97.5% respectively chose home as the place for care and death (*p* = 0.787). Patients who were followed up for longer were less likely to have specified their home as their preferred place for care or death, although this difference was not statistically significant (*p* = 0.404) (Appendix A.3).

### 3.4. Multivariate Analysis

The multivariate regression model was adjusted for age, sex, disease (oncological vs. non-oncological), having a primary caregiver, preference for death/care recorded as home and HPC follow-up (per week of follow-up). In the multivariable logistic regression model, a longer follow-up duration in the HPC program was independently associated with death at home (OR 0.952; 95% CI 0.919–0.986; *p* = 0.006) and with age. Female sex and the presence of a recorded preference as well as disease type, caregiver presence and area of residence were not significantly associated with the place of death (Table 3).

The overall model was statistically significant (*p* < 0.05), and the Hosmer–Lemeshow test indicated adequate model fit (χ^2^ = 9.892, df = 8, *p* = 0.273). The model explained 10% of the variance in clustering (Nagelkerke R^2^ = 0.10), Cox & Snell R^2^ = 0.06) and correctly classified 83% of the participants.

## 4. Discussion

This study examined factors associated with place of death among patients followed by an HPC team in an urban area of Madrid. Older age and female sex were associated with home death in the bivariate analyses, although these associations did not remain statistically significant in the multivariable model. Even though most patients with a recorded preference for home ultimately died at home, recording preferences was not independently associated with the place of death after adjustment. Although a high concordance between preferred and actual place of death was observed, this did not translate into an independent association after adjustment, suggesting that this concordance may be driven by underlying care processes rather than documentation itself. This indicates a limited explanatory capacity of the model, suggesting that relevant determinants of place of death were not fully captured by the variables included in the analysis. These findings suggest that documentation alone was not independently associated with place of death in this sample and should not be considered sufficient, in itself, to influence end-of-life outcomes. Any potential contribution of documentation must be interpreted within the broader context of care planning, caregiver support, and service availability.

The high proportion of home deaths observed in our study exceeds that reported in most population-based studies [4,5,6,7,8]. This discrepancy likely reflects the characteristics of patients referred to HPC teams, for whom a decision regarding end-of-life care location has often already been made prior to referral. In addition, access to specialized HPC and coordinated primary care may facilitate symptom control and prevent unnecessary hospital admissions.

Older age was associated with a greater probability of dying at home, a result that is consistent with the findings of previous studies [5,7,8,16]. This association may be explained by the greater acceptance by patients, families and healthcare providers of the appropriateness of focusing on comfort-oriented care rather than hospital transfer. Disease trajectories may also be more predictable in older patients, facilitating earlier palliative care involvement. Previous studies have shown that elderly patients with advanced illnesses prefer to die at home since they are closer to their loved ones, which alleviates the burden of symptomatology without increasing the burden on the caregiver, particularly when they are supported by services such as those provided by HPC teams and primary care doctors [12].

In our cohort, women were more likely to die at home than men were, although in the multivariate analysis it was not a statistically significant difference. Findings regarding sex differences in place of death are inconsistent in the literature [6,7,16,20], although some studies have shown that, similar to our findings, more women than men die at home [24]. Possible explanations for this sex difference include the greater involvement of women in ACP discussions and the avoidance of hospitalization. Moreover, women generally die at an older age, which may influence care decisions [8,24]. However, for a patient to die at home, the presence of an effective caregiver is necessary; owing to cultural influences, it is more likely that this role is played by a woman (wife, daughter, or sister), and thus, men are more likely to have caregivers. In addition to the potential influence of caregiver gender on the place of death, we consider that the relationship between follow-up duration and the likelihood of dying at home may also be shaped by the caregiver’s role. As the caregiving period becomes longer, caregivers are increasingly likely to experience burden and collapse, leading to a progressive depletion of their emotional reserves and making it more difficult to cope with the moment of death, particularly when the demands of care intensify over time. Consequently, longer follow-up intervals may reduce caregivers’ capacity to sustain home care, thereby indirectly influencing the probability of dying at home [35,36,37]. This interpretation should be considered hypothesis-generating, as caregiver-related variables were not directly measured in this study. However, information on caregiver characteristics was not available in our study and therefore should be addressed in future research, as the sex of the caregiver could play an important role in the place of death [38]. To this end, our study was conducted primarily in an urban environment in one of the largest cities in Europe, and it would be interesting to conduct further studies addressing caregiver characteristics [15,39].

We did not observe a significant association between the presence of a recorded caregiver and the place of death, in contrast to the findings of previous studies [13,15,17]. This discrepancy may be due to the underrecording or misclassification of this variable in routine clinical practice. However, our results may reflect a recording bias for this variable; in previously mentioned studies [15,38], having a primary caregiver positively influenced dying in the patient’s desired location.

Although ACP has been shown to be associated with increases in deaths at home [24,30], our findings do not support an independent association between documented preferences and place of death after controlling for additional covariates. Documentation in the clinical record should therefore be interpreted cautiously. It may reflect that goals-of-care conversations took place, but it does not capture the quality, timing, continuity, or scope of a full advance care planning process. In this sense, documentation should be understood as an operational proxy of ACP in routine practice rather than as equivalent to ACP itself. Honouring preferences likely depends on multiple interacting factors, including caregiver support, clinical evolution, service coordination, and resource availability. In addition, the multivariable model showed limited explanatory capacity, suggesting that relevant determinants of place of death were not captured by the variables included in the analysis. Similarly, it is important to expand the knowledge on how cultural differences can influence both the place of death and the preferences of each person, as well as the likelihood of starting ACP [3].

This study has several limitations. First, this study is limited by its retrospective design and the use of routinely collected data, which may introduce information bias and be affected by underrecording. Consequently, depending on the professional, certain information may be underreported. However, patients who prefer to die at home may state as such more clearly; therefore, there is an increased probability that this preference will be included in the clinical history, thus allowing their desires to be fulfilled more easily. As such, variability in the quality of the records—in terms of whether a patient has expressed his or her preference to the team or to his or her family or whether there has been a change in that preference and the professional has not recorded it—can be expected. In summary, the quality of the information available to us may be limited, resulting in information bias. Moreover, the presence of documentation in the clinical record cannot be assumed to represent a complete or formal ACP process, as it may only partially reflect prior discussions and may not capture changes in preferences over time.

Second, caregiver-related variables and socioeconomic factors were not included but may have influenced the place of death. Additionally, the study was conducted in a single urban area, which may limit generalisability. Although our study focused on a highly urbanized area and was not multicentric, it included individuals with a wide range of deprivation indices as measured through the MEDEA index [34], as it included individuals from areas with populations that have very different levels of education, or socioeconomic levels, providing greater strength to the data collected [5,7,17]. These unmeasured variables may partly explain the high rate of home deaths observed, independently of the preference recorded.

Third, the multivariable model had limited explanatory power, as reflected by the low Nagelkerke R^2^ value. This suggests that important determinants of place of death were not captured in the available dataset. Variables such as functional status, symptom burden, caregiver burden, family structure, social support, prior hospital use, patient complexity, and the intensity of community and health service involvement were not available and may have influenced both documentation of preferences and the actual place of death. Accordingly, the results should be interpreted as exploratory associations rather than as a comprehensive explanation of the observed outcomes.

A major strength of this study is the use of real-world data from a large and heterogeneous population, enhancing the external validity for similar urban settings. Similarly, the study had a large and heterogeneous sample size that, by exhibiting a relatively balanced distribution between the groups, supports its external validity and favours its translation to other care contexts.

In this retrospective observational study with an exploratory approach, older age and female sex were associated with home death in bivariate analyses, although these associations did not remain statistically significant after adjustment. Although most patients with a documented preference for home died at home, documentation of the preferred place of care or death was not independently associated with place of death in the multivariable analysis. These findings should be interpreted cautiously, given the limited explanatory power of the model and the absence of potentially relevant unmeasured variables. Documentation of preferences remains clinically relevant as part of patient-centred care and advance care planning, but it should not be interpreted as sufficient in itself to determine end-of-life outcomes. Further studies including a broader range of clinical, social, and caregiver-related variables are needed.

## 5. Conclusions

In clinical practice, systematically exploring and documenting patients’ preferences regarding place of care and death may support patient-centred decision-making and help align care with patients’ values. However, achieving the preferred place of death likely depends on a complex interplay of clinical, social, and care-related factors. Documentation alone should therefore not be interpreted as independently influencing the place of death, as outcomes are likely driven by multiple interacting factors beyond documentation itself. Rather, it should be understood as one component within a broader advance care planning process that requires adequate resources, caregiver support, and coordinated care.

## Figures and Tables

**Table 1:** Baseline characteristics of the study population (n = 464). Abbreviations: SD, standard deviation; IQR, interquartile range.

		n = 464	%
Sex (Female)		246	53
Had a caregiver (Yes)		112	24.1
Age (years, SD)		80.8 ± 13.0	
HPC follow-up duration (weeks, median, IQR)		2.86 (1–8.14)	
Place of death	Home	383	82.5
	Hospital	65	14.1
	Mid-stay Palliative Care Unit	16	3.4
Preferred place of death	Not recorded	350	75.4
	Home	109	23.5
	Hospital	5	1.1
Preferred place of care or death	Not recorded	167	36
	Home	290	62.5
	Hospital	5	1.1
	Mid-stay Palliative Care Unit	2	0.4

Notes: SD: standard deviation, HPC: home palliative care, IQR: interquartile range. “Unrecorded” indicates the absence of documentation of a preference in the clinical record and should not be interpreted as a stated preference category.

**Table 2:** Variables associated with death at home (bivariate analysis).

Variable	Home Death n (%)	Non-Home Death n (%)	*p* Value	x^2^ or *t*
Male sex	171 (78.4%)	47 (21.6%)	0.037	1.714 *
Female sex	212 (86.2%)	34 (13.8%)		
Age (mean, SD)	81.49 (12.8)	77.70 (13.5)	0.017	2.399 ′
Weeks in program (mean, SD)	6.89 (12.2)	11.1 (13.2)	<0.01	2.658 ″
Non-oncological disease	156 (87.2%)	23 (12.8%)	0.04	4.294 *
Oncological disease	227 (79.6%)	58 (20.4%)		
Primary caregiver	296 (84.1%)	56 (15.9%)	0.15	2.425 *
No caregiver	87 (77.7%)	25 (22.3%)		
Mixed preference regarding place of care and death recorded	248 (83.5%)	49 (16.5%)	0.273	0.526 *
Mixed preference regarding place of care and death not recorded	135(80.5%)	32 (19.2%)		
Preference for home as place of death	242 (83.4%)	48 (16.6%)	0.675	0.025 *
Preference for clinical setting as place of death	6 (85.7%)	1 (14.3%)		

* Chi^2^, ′ T student, ″ Welch.

**Table 3:** Multivariable logistic regression for death at home. Abbreviations: OR, odds ratio; CI, confidence interval (n = 297).

Variable	Adjusted OR (95% CI)	*p* Value
Age (per year increase)	1.025 (1.001–1.050)	0.038
Female sex (vs. male)	1.588 (0.833–3.026)	0.160
Area of residence	1.030 (0.770–1.379)	0.842
Non-oncological disease (vs. oncological)	0.851 (0.388–1.864)	0.686
HPC follow-up duration (per week of follow-up)	0.952 (0.919–0.986)	0.006
Primary caregiver (yes vs. no)	0.431 (0.431–1.627)	0.600
Home as Preferred of place of death (yes vs. no)	0.561 (0.059–5.356)	0.616

Notes: The model was adjusted for age, sex, type of disease, household quartile income area, primary caregiver, HPC follow-up duration and preference for death. Abbreviations: OR: odds ratio; CI: confidence interval; and HPC: home palliative care.

## Data Availability

The datasets generated and analysed during the current study are not publicly available because of privacy restrictions but can be obtained upon reasonable request.

## Abbreviations

AECC	Asociación Española Contra el Cáncer (Spanish Association Against Cancer)
PC	Palliative care
HPC	Home palliative care
AD	Alzheimer’s disease
ALS	Amyotrophic lateral sclerosis
MS	Multiple sclerosis
COPD	Chronic obstructive pulmonary disease
ACP	Advance care planning
SERMAS	Servicio Madrileño de Salud (Madrid Health Service)
MSPCU	Mid-stay palliative care unit
APCU	Acute palliative care hospitalization unit

## Appendix A

### Appendix A.1. Clinical Characteristics of the Participants

**Group**	**Subgroup**	**% in** **Subgroup**	**Pathology**	**N**	**%**
Non-oncological	Neurodegenerative diseases	20.7	Alzheimer’s disease (AD)	8	1.72
			Parkinson’s disease	3	0.65
			Dementia with Lewy bodies	1	0.22
			Prion disease	1	0.22
			Amyotrophic lateral sclerosis (ALS)	5	1.08
			Multiple sclerosis (MS)	2	0.43
			Other dementias/neurodegenerative diseases	17	3.66
	Respiratory diseases	17.8	Chronic obstructive pulmonary disease (COPD)	15	3.23
			Interstitial lung diseases	6	1.29
			Other chronic respiratory diseases	11	2.37
	Organ deficiencies	28.5	Heart failure	34	7.33
			Chronic kidney disease	13	2.8
			Hepatic insufficiency	4	0.86
	Vascular diseases	5.6	Stroke	7	1.51
			Other	3	0.65
	Other non-oncological diseases	27.4	Advanced geriatric disease	31	6.68
			Other	18	3.88
Oncological	Digestive	36.9	Colon cancer	37	7.97
			Rectal cancer	13	2.8
			Pancreatic cancer	20	4.31
			Cholangiocarcinoma	5	1.08
			Hepatocellular carcinoma	8	1.72
			Oesophageal cancer	2	0.43
			Gastric cancer	1	0.22
			Other digestive cancers	19	4.09
	Respiratory	17.9	Bronchus and lung cancer	51	10.99
	Gynaecological	15.8	Breast cancer	24	5.17
			Ovarian cancer	12	2.59
			Endometrial, cervical, and uterine body cancers	8	1.72
			Vulvar cancer	1	0.22
	Genitourinary	12.9	Bladder cancer	14	3.02
			Prostate cancer	13	2.8
			Renal cancer	9	1.94
			Other genitourinary cancers	1	0.22
	Haematological	3.2	Multiple myeloma	4	0.86
			Lymphomas	3	0.65
			Acute myeloid leukaemia	2	0.43
	Skin and appendages	2.8	Melanoma	7	1.51
			Basal cell carcinoma	1	0.22
	Head and neck	2.5	Face, head and neck cancers	7	1.51
	Central nervous system	1.4	Glioblastoma multiforme	4	0.86
	Bone and soft tissue	0.7	Liposarcoma	2	0.43
	Other oncological diseases	5.9	Other cancers	17	3.66
Total				464	100

### Appendix A.2. Factors Associated with Recording Preferences

**Variable**	**Preferred Place of Care or Death Recorded n (%)** **N = 297 (64.0%)**	**Preferred Place of Care or Death NON** **Recorded n (%)** **n = 167 (36.0%)**	***p* Value**
Male sex	142 (47.81%)	76 (45.51%)	0.633
Female sex	155 (52.19%)	91 (54.49%)	
Age (mean, SD)	79.93 (13.67)	82.44 (11.53)	0.036 *
Non-oncological disease	103 (34.68%)	76 (45.51%)	0.021
Oncological disease	194 (65.32%)	91 (54.49%)	
Area of residence			0.821
1	83 (64.8%)	45 (35.2%)	
2	68 (66.0%)	35 (34.0%)	
3	64 (65.3%)	34 (34.7%)	
4	82 (60.7%)	53 (39.3%)	
Primary caregiver			0.001
Yes	99 (88.4%)	13 (11.6%)	
No	198 (56.3%)	154 (43.7%)	
HPC follow-up duration (per week of follow-up) (mean, SD)	5.37 (7.70)	11.64 (17.38)	0.001
* Difference in means 2.52 (IC 95%: 0.17–4.86).			

### Appendix A.3. Factors Associated with Preferences Expressed

**Variable**	**Home as Preferred Place of Care or Death (%)** **N = 290 (97.6%)**	**Non-Home as Preferred Place of Care or Death (%)** **n = 7 (2.4%)**	***p* Value**
Male sex	136 (95.8%)	6 (4.2%)	0.042
Female sex	154 (99.4%)	1 (0.6%)	
Age (mean, SD)	79.87 (13.71)	82.14 (12.89)	0.661 *
Non-oncological disease	102 (99.0%)	1 (1.0%)	0.251
Oncological disease	188 (96.9%)	6 (3.1%)	
Area of residence			0.522
1	81 (97.6%)	2 (2.4%)	
2	66 (97.1%)	2 (2.9%)	
3	64 (100.0%)	0 (0.0%)	
4	79 (96.3%)	3 (3.7%)	
Primary caregiver			0.787
Yes	97 (98.0%)	2 (2.0%)	
No	193 (97.5%)	5 (2.5%)	
HPC follow-up duration (per week of follow-up) (mean, SD)	5.27 (7.54)	9.63 (12.81)	0.404 ″
* Difference in means 2.27 (IC 95%: −9.66–14.20). ″ Difference in means 4.36 (IC 95%: −7.49–16.21).			
